# Increased Attentional Bias Toward Visual Cues in Internet Gaming Disorder and Obsessive-Compulsive Disorder: An Event-Related Potential Study

**DOI:** 10.3389/fpsyt.2018.00315

**Published:** 2018-07-13

**Authors:** Sung Nyun Kim, Minah Kim, Tak Hyung Lee, Ji-Yoon Lee, Sunyoung Park, Minkyung Park, Dai-Jin Kim, Jun Soo Kwon, Jung-Seok Choi

**Affiliations:** ^1^Department of Psychiatry, Seoul Medical Center, Seoul, South Korea; ^2^Department of Neuropsychiatry, Seoul National University Hospital, Seoul, South Korea; ^3^Department of Brain and Cognitive Science, Seoul National University College of Natural Science, Seoul, South Korea; ^4^Department of Psychiatry, SMG-SNU Boramae Medical Center, Seoul, South Korea; ^5^Department of Psychiatry, Seoul St. Mary's Hospital, The Catholic University of Korea College of Medicine, Seoul, South Korea

**Keywords:** event-related potential, late positive potential, craving, attentional bias, internet gaming disorder, obsessive compulsive disorder, cue reactivity

## Abstract

Internet gaming disorder (IGD) is a newly identified potential addiction disorder associated with compulsive internet-game playing behavior and attentional bias toward online gaming- related cues. Attentional bias toward addiction-related cues is the core feature of addiction that is associated with craving, but the pathophysiology of attentional bias in IGD is not well-understood, such as its relationship to compulsivity. In this study, we used the electrophysiological marker of late positive potential (LPP) to compare attentional bias in IGD and obsessive compulsive disorder (OCD). Twenty patients with IGD, 20 patients with OCD, and 23 healthy control (HC) subjects viewed a series of game-related, OCD-related, and neutral pictures while their event-related potentials (ERPs) were recorded. The game-related cues included in-game screen captures of popular internet games. The OCD-related cues included pictures which provokes obsessive and compulsive symptoms of contamination/washing or checking. LPPs were calculated as the mean value of amplitudes between 350 and 750 ms at the centro-parietal (CP1, CPz, CP2) and parietal (P1, Pz, P2) electrode sites. Higher LPP amplitudes were found for game-related cues in the IGD group than in the HCs, and higher LPP amplitudes were observed in the OCD group for OCD-related cues. The IGD group did not exhibit LPP changes in response to OCD-related cues. Subjective scales demonstrated increased arousal to game-related cues and OCD-related cues in both the IGD and OCD groups compared with the HC group. Increased LPPs in response to disorder-specific cues (game-related and OCD-related) were found in both IGD and OCD groups respectively, although the groups showed overlapping arousal on subjective scales. Our results indicate that LPP is a candidate neurophysiological marker for cue-related craving in IGD.

## Introduction

Internet gaming disorder (IGD) was newly included as a putative addiction disorder in section Results, or “a condition for further study” of the Diagnostic and Statistical Manual of Mental Disorders, 5th edition (DSM-5) (1). The DSM-5 defines the condition as “excessive and prolonged pattern of internet gaming leading to clinically significant impairment or distress.” The symptoms of IGD are similar to those of addiction-related phenomenon, including “continued excessive use of internet games despite adverse consequences,” “loss of control (compulsive playing),” and “craving” (2, 3). Accordingly, many researchers proposed that IGD be regarded as one of behavioral addictions. However, there are concerns of other researchers that current operationalization of IGD criteria needs more specificity, because the problematic behaviors of pathologic gaming may be different from those of substance use disorders (4). Therefore integrative understanding of neurobiological substrate and clinical phenomenon of IGD is important to clarify the condition from other disorders with similar clinical features.

One of core phenomenology of addiction is repeated behavior involving continued excessive use of substance or behavior. Obsessive-compulsive disorder (OCD) is also related to repetitive compulsive behavior, so there is some phenomenological overlap between IGD and OCD in terms of repetitive behaviors (5). Traditionally, repetitive behaviors can be seen in terms of the domain of impulsivity and compulsivity, where impulsivity and compulsivity have been proposed as opposite constructs. Impulsivity is described as a predisposition toward unplanned reactions to stimuli, meanwhile compulsivity involves repetitive behavior often motivated by the need to reduce or prevent anxiety (6). Previous conceptualizations of IGD have focused on the behavioral feature of impulsivity (7). However in a recent study, directly comparing impulsivity and compulsivity in IGD, OCD, and alcohol use disorder patients using neurocognitive measurements, IGD seemed to share neurocognitive dysfunctions of impulsivity and compulsivity (5). In addition, neurobiological studies have demonstrated the dysfunction of frontal areas within inhibitory brain circuitry or reward circuitry in addiction, which may be related to both impulsivity and compulsivity (8, 9). A recent review proposed IGD lie between behavioral addiction and impulse-control disorders as initial impulsivity followed by compulsivity in behavioral addiction can be differentiated from impulse-control disorder (10). Taken together, direct comparison of IGD and OCD with neurobiological substrate may provide more specific conceptualization between impulsivity and compulsivity.

Craving is another of the core characteristics of addiction (11). Conditioning to drug-related cues underlies the change in salience, and the change in mesolimbic dopamine transmission is regarded as the mechanism. Attentional bias is enhanced attention being afforded to drugs and drug-related cues due to increased incentive salience (12), is often used to examine craving (13, 14). Attentional bias is associated with cue-elicited craving. In IGD, the brain regions activated in response to the visual presentation of gaming cues correspond to the key regions associated with drug-related addiction (15). A previous study demonstrated that the efficacy of treatment for IGD is associated with changes in functional connectivity in the cortical-ventral striatum circuitry, which mediates gaming craving and attentional bias (16). Several previous studies have employed the late positive potential (LPP) and event-related potential (ERP) components in the study of attentional bias, which represent motivated attention to emotionally salient stimuli (17, 18). The LPP is a midline ERP that becomes evident approximately from 350 to 750 ms following arousing emotional stimulus (17, 19). Increased LPP amplitude in response to drug-related cues has consistently been observed in various substance use disorders, such as cocaine use, alcohol use disorder and smoking (20–22). Recently, a few studies involving individuals with OCD have reported abnormalities in LPP in response to OCD-related pictures; however, only one study showed a stronger late positive complex, which is similar to LPP, in excessive computer game players compared to casual players using parietal electrodes (23).

The aim of this study was to examine and compare cue-related reactivity in individuals with IGD and OCD using LPP. We hypothesized that individuals with IGD would exhibit increased LPPs in response to game-related images. In addition, we aimed to compare the cue reactivity in IGD patients as well as the OCD patients.

## Methods

### Participants and clinical assessments

Twenty patients with IGD, 20 patients with OCD, and 23 healthy control (HC) subjects participated in this study. The participants in the IGD and HC groups were recruited from the addiction outpatient clinic at the SMG-SNU Boramae Medical Center and via an internet advertisement. The participants in the OCD group were recruited from the OCD outpatient clinic at Seoul National University Hospital (SNUH). An experienced psychiatrist conducted interviews to confirm the diagnoses of IGD and OCD using the DSM-5 criteria. OCD patients with comorbid IGD were excluded from this study. All subjects with IGD were drug-naïve and participated in one of three popular internet games (League of Legend, FIFA, Sudden Attack), which were selected as game-related cues, for >4 h/day. Only patients with OCD who had compulsive symptoms of the washing or checking type were included, as pictures provoking washing or checking compulsions were selected as OCD-related cues. Six of the OCD patients were medication-naïve, 8 were medication-free for >1 month before entering the study, and 6 were medicated at the time of testing. The six medicated OCD patients were taking selective serotonin reuptake inhibitors, and one patient was prescribed a small dose of olanzapine (2.5 mg) as an adjuvant. The HC subjects played internet games for <2 h/day and were confirmed to have no past or current psychiatric illness using the Mini-International Neuropsychiatric Interview (24). For all the participants, Young's Internet Addiction Test (IAT) (25) was used to measure the severity of internet gaming addiction, and the severity of OCD was assessed using the Yale-Brown obsessive compulsive scale (Y-BOCS) (26). The Hamilton rating scale for depression (HAM-D) (27) and the Hamilton rating scale for anxiety (HAM-A) (28) were used to assess depressive and anxiety symptoms. The participants' intelligence quotient (IQ) was measured using the abbreviated version of the Korean-Wechsler Adult Intelligence Scale. The exclusion criteria included a lifetime diagnosis of substance abuse or dependence, neurological disease, significant head injury accompanied by loss of consciousness, any medical illness with documented cognitive sequelae, sensory impairments, and intellectual disability (IQ < 70).

All the participants fully understood the study procedure and provided written informed consent. The study was conducted in accordance with the Declaration of Helsinki, and the institutional review boards of SMG-SNU Boramae Medical Center and SNUH approved the study.

### Cue reactivity task and EEG recordings

**Figure 1:**
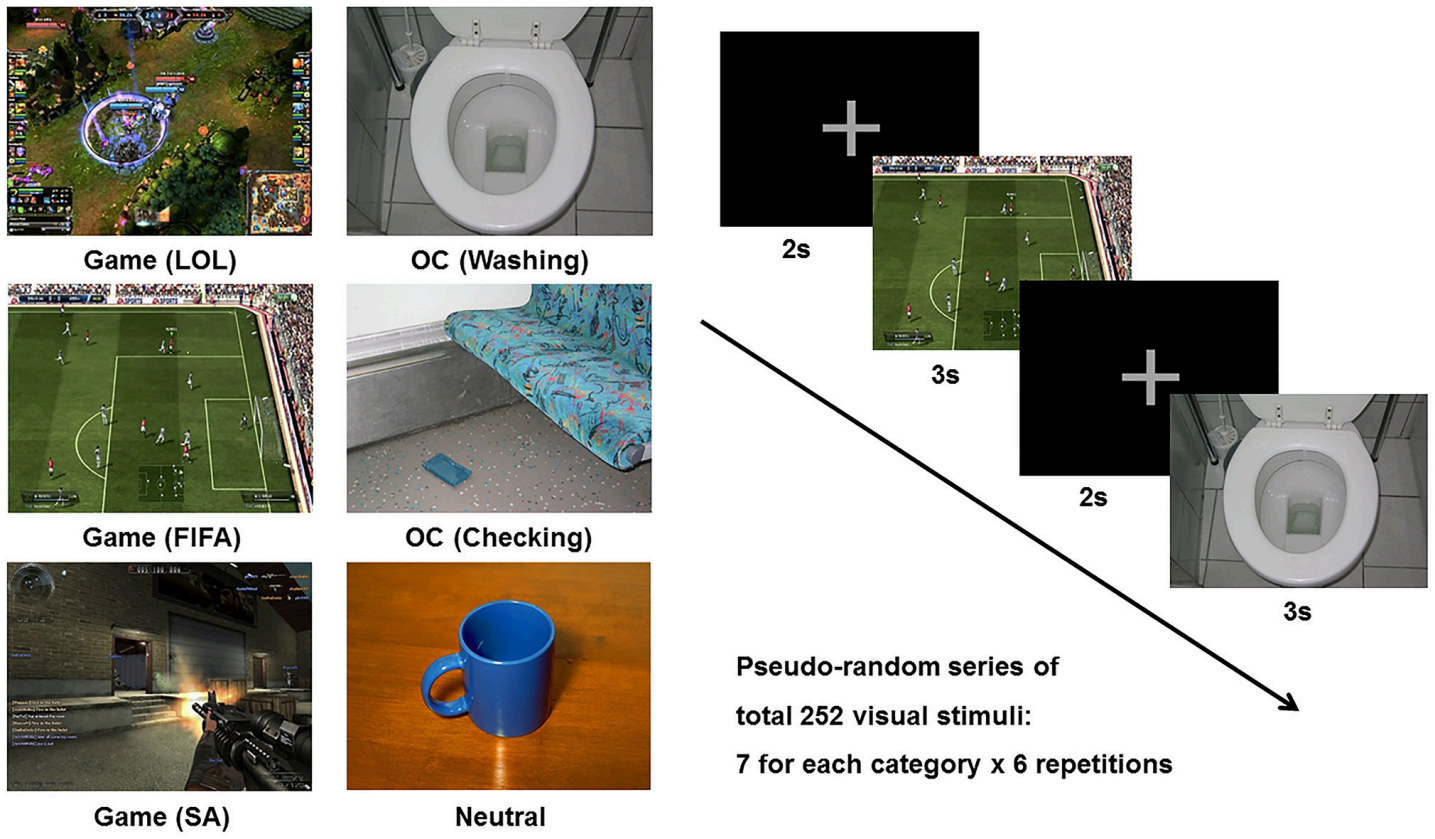
Example of the six categories of stimuli (League of Legend, FIFA, Sudden Attack, Washing, Checking, Neutral) and a sample task sequence. Game, game-related cue; LOL, League of Legend; SA, Sudden Attack; OCD, obsessive-compulsive disorder-related cue.

The cue reactivity task consisted of three types of picture sets: (1) the game-related cues included in-game screen captures of three popular internet games (i.e., *League of Legends, FIFA*, and *Sudden Attack*); (2) the OCD-related cues included pictures from the contamination/washing and checking categories in the Berlin Obsessive Compulsive Disorder Picture Set (BOCD-PS), which were validated for provocation of anxiety and compulsive behavior (29); and (3) the neutral pictures were adopted from the neutral category of the International Affective Picture System (IAPS) (30). Each individual visual stimulus was matched for size (resolution of 1,024 × 768 pixels, 361 × 271 mm, 72 dpi), luminance, brightness, and color. Each category (League of Legends, FIFA, Sudden Attack, Washing, Checking, Neutral) consisted of 7 different pictures, and a pseudo-random series of pictures was repeated 6 times during the task run. The stimulus duration was 3,000 ms, and the inter-stimulus interval was 2,000 ms. The six categories of picture stimuli and sample task sequences are presented in Figure 1.

The participants were seated comfortably in a dimly lit, electrically shielded room ~60 cm from a monitor and instructed to watch the screen carefully. Continuous electroencephalogram (EEG) recordings were made using a Neuroscan 128-channel Synamps system with a 128-channel Quick-Cap based on the modified 10–20 international system (Compumedics, Charlotte, NC). The electrodes at the mastoid sites served as reference electrodes, and the ground electrode was placed between the FPz and Fz electrode sites. The EEG was digitized at a 1,000-Hz sampling rate with an online filter of 0.05–100 Hz. Eye-movement artifacts were monitored by recording the vertical and horizontal electro-oculogram using electrodes below and on the outer canthus of the left eye. The resistance at all the electrode sites was below 5 kΩ. After completing the EEG recording during the cue reactivity task, the participants were asked to rate their arousal and valence for all stimuli, their craving in response to the game-related cues, and their desire to engage in compulsive behavior (compulsion) for the OCD-related cues using a visual analog scale ranging from 0 to 10.

### ERP analysis

The ERP data were pre-processed using Curry 7.0 software (Compumedics, Charlotte, NC). The EEG recordings were re-referenced to a common average reference, and eye-movement artifacts were reduced using the artifact-reduction algorithm in the Curry software (31). The continuous EEG data were bandpass filtered between 0.1 and 30 Hz, epoched to 200 ms pre-stimulus and 3,000 ms post-stimulus, and baseline-corrected using the averaged pre-stimulus interval voltage. Epochs containing EEG amplitudes that exceeded ± 100 μV were rejected automatically. The epochs were then averaged separately for each class (Game vs. OCD vs. Neutral). The LPPs were calculated as the mean values of amplitudes between 350 and 750 ms at the centro-parietal (CP1, CPz, CP2) and parietal (P1, Pz, P2) electrode sites. Electrode sites were selected as reported in a previous study (23).

### Statistical analysis

Demographics, clinical characteristics, and rating scores were compared among the groups using one-way analysis of variance (ANOVA), independent sample *t-*tests, or Welch's test if the variances were not equal. A χ^2^ analysis or Fisher's exact test was used for categorical data analysis. The cue effects on the mean LPP amplitudes were analyzed using repeated-measures ANOVAs with the electrode sites (CP1, CPz, CP2, P1, Pz, P2) and the 3 stimuli (Game, OCD, Neutral) as within-subject factors and group (HC, IGD, OCD) as a between-subjects factor. Group comparisons of the mean LPP amplitudes were performed using repeated-measures ANOVAs with the 6 centro-parietal electrode sites as the within-subject factors and group (HC, IGD, OCD) as a between-subjects factor. A *post-hoc* Tukey's honestly significant difference (HSD) test was used to identify specific group differences. SPSS software (ver. 22.0; IBM Corp., Armonk, NY) was used for the statistical analyses. Statistical significance was set at *P* < 0.05.

## Results

### Demographics, clinical characteristics, and ratings for each stimulus

We did not find significant differences with regard to sex, handedness, IQ, or education (Table 1). However, all clinical characteristics (i.e., scores on the IAT, Y-BOCS, HAM-D, and HAM-A) exhibited significant group differences. The participants with IGD had the highest scores on the IAT, the patients with OCD had intermediate scores, and the HCs had the lowest scores on the IAT (IGD vs. HC, *P* < 0.001; IGD vs. OCD, *P* < 0.001; OCD vs. HC, *P* = 0.425). The patients with OCD had higher Y-BOCS, HAM-D, and HAM-A scores than the subjects with IGD (*P* < 0.001) and the HCs (*P* < 0.001). Table 2 summarizes the results of the ratings for arousal, valence, craving, and compulsion elicited by each stimulus category. The game-related cues elicited higher arousal in the patients with IGD and the patients with OCD than in the HC subjects (IGD vs. HC, *P* < 0.001; OCD vs. HC, *P* = 0.020). The OCD-related cues also provoked increased arousal among the patients with IGD and those with OCD compared with the level of arousal reported by HC subjects (IGD vs. HC, *P* = 0.012; OCD vs. HC, *P* < 0.001). The patients with OCD exhibited significantly higher arousal levels to the neutral IAPS pictures than did the HC group (*P* < 0.001). Valence did not differ across the groups for any of the three stimulus categories.

**Table 1:** Demographic and clinical characteristics of participants.

	**HC**	**IGD**	**OCD**	**Statistical analysis**^**a**^	
	**(*N* = 23)**	**(*N* = 20)**	**(*N* = 20)**	**χ^2^ or *F***	***P***
Sex (Male/Female)	15/8	19/1	15/5	5.621	0.060
Handedness (Right/Left)	22/1	18/2	20/0	2.219	0.330
Age (years)	24.8 (4.7)	24.5 (4.2)	25.3 (6.1)	0.145	0.865
IQ	116.2 (8.0)	108.1 (15.2)	109.3 (15.6)	2.451	0.098
Education (years)	13.7 (1.3)	14.5 (1.7)	13.7 (2.0)	1.503	0.231
IAT^b^	32.7 (11.4)	61.5 (11.8)	37.2 (10.8)	38.319	<0.001^**^
Hours of game playing	0.4 (0.6)	4.4 (3.4)	1.0 (1.5)	20.663	<0.001^**^
Y-BOCS Total	0.0 (0.2)	1.1 (2.3)	21.4 (6.7)	183.801	<0.001^**^
Obsession	0.0 (0.0)	0.5 (1.5)	10.7 (4.1)	124.357	<0.001^**^
Compulsion	0.0 (0.2)	1.0 (2.1)	10.7 (3.1)	164.675	<0.001^**^
HAM-D	2.2 (3.0)	5.2 (4.1)	12.0 (6.5)	23.626	<0.001^**^
HAM-A	1.2 (1.4)	3.5 (2.9)	11.9 (7.0)	34.427	<0.001^**^

HC, healthy control; IGD, internet gaming disorder; OCD, obsessive-compulsive disorder; IQ, intelligence quotient; IAT, Korean version of Young's Internet Addiction Test; Y-BOCS, Yale-Brown obsessive-compulsive scale; HAM-D, Hamilton rating scale for depression; HAM-A, Hamilton rating scale for anxiety.

Data are presented as means (standard deviation).

** P < 0.005.

a Analysis of variance, χ^2^ analysis or Fisher's exact test for categorical data.

b *With 2 missing values in OCD group*.

**Table 2:** Ratings for arousal, valence, craving, and compulsion elicited by each stimulus category.

	**HC**	**IGD**	**OCD**	**Statistical analysis**^**a**^		***Post-hoc*** **analysis**		
	**(*N* = 22)**	**(*N* = 20)**	**(*N* = 20)**	***F***	***P***	**IGD vs. HC**	**OCD vs. HC**	**IGD vs. OCD**
**GAME STIMULI (LOL, FIFA, SA)**								
Arousal^b^	2.7 (1.7)	4.7 (1.6)	4.1 (1.3)	8.934	<0.001^**^	<0.001^**^	0.020^*^	0.385
Valence^c^	4.7 (0.8)	5.3 (0.9)	4.9 (1.3)	1.655	0.200	0.183	0.851	0.449
Craving^d^	2.6 (1.9)	4.6 (1.8)	3.7 (1.5)	6.967	0.002^**^	0.001^**^	0.107	0.246
**OCD-RELATED STIMULI (WASHING, CHECKING)**								
Arousal^b^	2.8 (1.3)	4.1 (1.4)	4.8 (1.5)	10.855	<0.001^**^	0.012^*^	<0.001^**^	0.272
Valence^c^	3.8 (0.5)	3.7 (0.6)	3.5 (1.1)	0.978	0.382	0.885	0.357	0.649
Compulsion^e^	3.3 (1.5)	4.4 (1.6)	5.1 (1.8)	6.398	0.003^**^	0.067	0.002^**^	0.456
**NEUTRAL STIMULI (IAPS NEUTRAL)**								
Arousal^b^	2.0 (0.8)	2.7 (1.4)	3.5 (1.3)	8.591	0.001^**^	0.164	<0.001^**^	0.072
Valence^c^	5.8 (0.9)	5.6 (0.7)	5.5 (0.9)	0.932	0.399	0.612	0.392	0.931

HC, healthy control; IGD, internet gaming disorder; OCD, obsessive-compulsive disorder; LOL, League of Legend; SA, Sudden Attack; IAPS, International Affective Picture System.

Data are presented as means (standard deviation).

* P < 0.05.

** P < 0.005.

a Analysis of variance.

b Arousal: extreme calmness (0) ~ extreme excitement (10).

c Valence: extremely negative (0) ~ extremely positive (10).

d Craving: no desire to play game (0) ~ extreme desire to play game (10).

e *Compulsion: no desire to engage in compulsive behavior (0) ~ extreme desire to engage in compulsive behavior (10)*.

### LPP amplitudes

**Figure 2:**
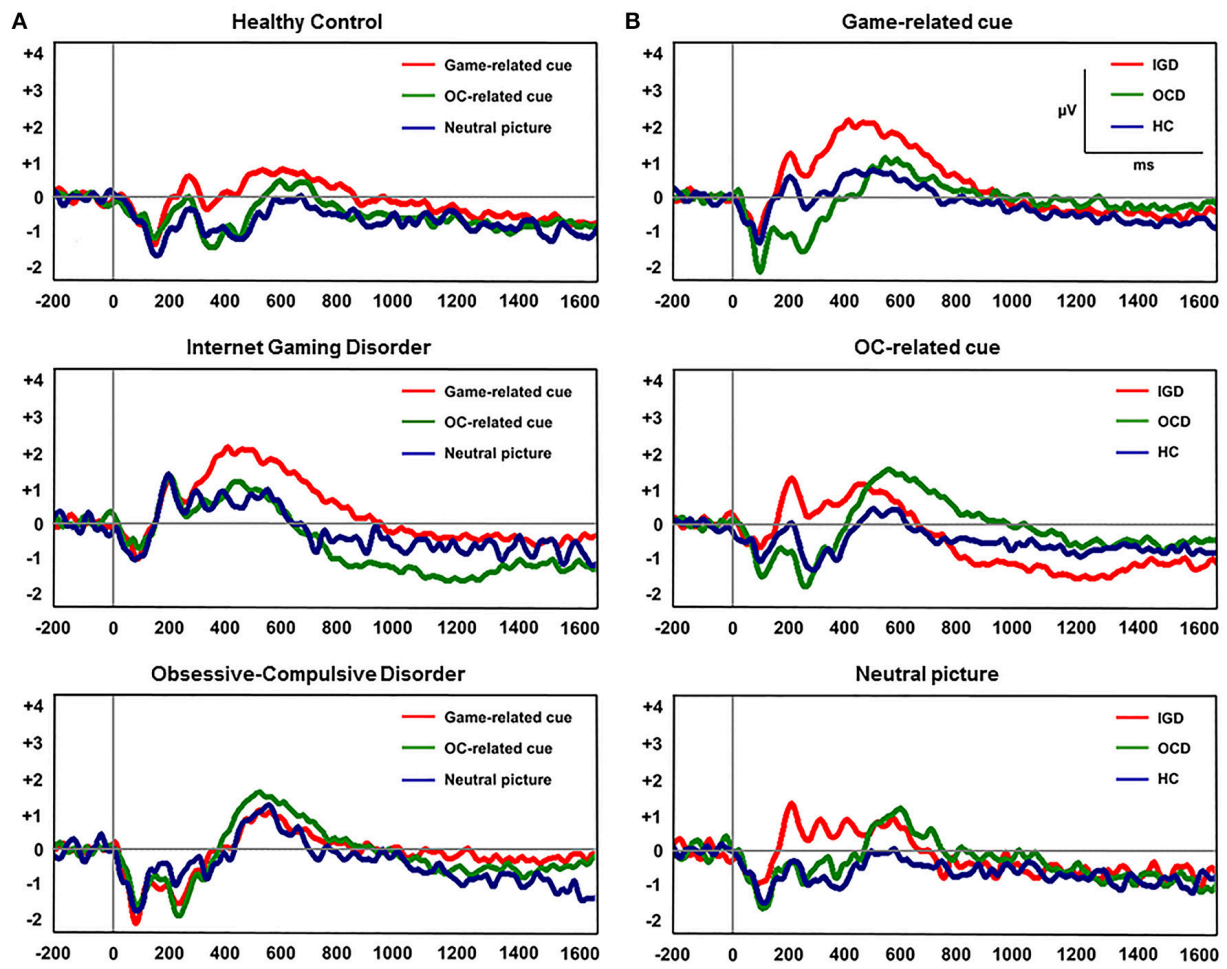
**(A)** Grand-averaged late positive potential (LPP) waveforms elicited by the three classes of cue-related pictures at the CPz electrode site. **(B)** Grand-averaged LPP waveforms across the three participant groups at the CPz electrode site.

Figure 2 displays the grand-averaged LPP waveforms at CPz across the three participant groups and the three cue categories. A significant main effect of cue (Game, OCD, Neutral) on mean LPP amplitude [*F*_(1, 60)_ = 11.298, *P* < 0.001] and group by cue interaction [*F*_(1, 60)_ = 3.957, *P* = 0.005] was found. Repeated-measures ANOVAs with the electrode sites (6 centro-parietal electrode sites) as within-subject factors and group (HC, IGD, OCD) as a between-subjects factor revealed a significant main effect of group on mean LPP amplitude for game-related cues [*F*_(2, 60)_ = 3.732, *P* = 0.022] and OCD-related cues [*F*_(2, 60)_ = 3.739, *P* = 0.029]. Group effect was not significant for the mean LPP amplitude elicited by the neutral IAPS pictures [*F*_(2, 60)_ = 0.574, *P* = 0.566]. A *post-hoc* Tukey's HSD test demonstrated that the mean LPP amplitude for game-related cues was enhanced in the subjects with IGD (*P* = 0.022) compared with that in the HCs. In addition, the mean LPP amplitude for the OCD-related cues was higher in the patients with OCD (*P* = 0.029) than in the HC subjects. Table 3 presents the means (standard deviations) and the group comparison results for LPP amplitude at each electrode site. The game-related cues elicited higher LPP amplitudes in the patients with IGD than in the HC subjects at the CP1, CPz, and P2 electrode sites. At the CP2 electrode site, the patients with OCD exhibited increased LPP amplitude compared with the patients with IGD. The subjects with OCD exhibited higher LPP amplitudes than the HC subjects at the P2 electrode site.

**Table 3:** Comparison of late positive potentials (LPPs) that averaged between 350 and 750 ms post-stimulus onset across the three groups.

**Late positive potential (μV)**	**HC**	**IGD**	**OCD**	**Statistical analysis**^**a**^		***Post-hoc*** **analysis**		
	**(*N* = 23)**	**(*N* = 20)**	**(*N* = 20)**	***F***	***P***	**IGD vs. HC**	**OCD vs. HC**	**IGD vs. OCD**
**GAME STIMULI (LOL, FIFA, SA)**								
CP1 electrode site	0.3 (1.3)	1.6 (1.6)	1.1 (1.7)	4.253	0.019^*^	0.015^*^	0.193	0.527
CPz electrode site	−0.2 (1.4)	1.2 (2.1)	0.3 (1.6)	3.370	0.041^*^	0.034^*^	0.671	0.230
CP2 electrode site	0.4 (1.2)	0.6 (2.1)	1.0 (1.9)	0.668	0.516	0.932	0.494	0.731
P1 electrode site	2.5 (1.8)	3.6 (2.5)	2.7 (1.2)	1.857	0.165	0.164	0.930	0.331
Pz electrode site	2.4 (1.8)	3.4 (1.9)	2.8 (2.0)	1.485	0.235	0.205	0.730	0.622
P2 electrode site	2.3 (1.6)	3.8 (1.8)	3.1 (2.2)	3.525	0.036^*^	0.027^*^	0.347	0.455
**OCD-RELATED STIMULI (WASHING, CHECKING)**								
CP1 electrode site	0.2 (1.4)	0.5 (1.6)	1.3 (1.5)	2.742	0.073	0.857	0.069	0.222
CPz electrode site	−0.3 (1.1)	0.1 (2.3)	0.6 (2.3)	1.290	0.283	0.820	0.254	0.602
CP2 electrode site	0.5 (1.5)	−0.5 (1.4)	1.3 (2.1)	5.744	0.005^*^	0.149	0.251	0.004^**^
P1 electrode site	2.5 (2.2)	2.7 (2.3)	3.1 (1.3)	0.509	0.603	0.942	0.581	0.795
Pz electrode site	2.2 (1.8)	2.6 (1.8)	3.4 (1.5)	2.444	0.095	0.716	0.080	0.365
P2 electrode site	2.2 (1.7)	3.2 (2.0)	3.6 (1.9)	3.528	0.036^*^	0.190	0.033^*^	0.713
**NEUTRAL STIMULI (IAPS NEUTRAL)**								
CP1 electrode site	0.0 (1.4)	0.3 (1.4)	0.5 (1.9)	0.578	0.564	0.776	0.546	0.931
CPz electrode site	−0.5 (1.5)	0.1 (2.0)	−0.1 (1.7)	0.673	0.514	0.500	0.728	0.933
CP2 electrode site	0.1 (1.5)	−1.0 (1.6)	0.4 (1.7)	2.595	0.083	0.230	0.814	0.080
P1 electrode site	2.2 (2.1)	2.3 (2.2)	1.3 (0.3)	0.169	0.845	0.956	0.947	0.831
Pz electrode site	1.9 (2.0)	2.3 (1.6)	2.4 (1.6)	0.536	0.588	0.759	0.581	0.958
P2 electrode site	2.0 (1.9)	2.7 (2.0)	2.8 (2.0)	1.313	0.277	0.410	0.311	0.982

HC, healthy control; IGD, internet gaming disorder; OCD, obsessive-compulsive disorder; LOL, League of Legend; SA, Sudden Attack; IAPS, International Affective Picture System.

Data are presented as means (standard deviation).

* P < 0.05.

** P < 0.005.

a *Analysis of variance*.

### Correlations

We could not find any significant correlation between LPPs and clinical scales, such as severity of addiction in the IGD group and YBOCS scores among OCD patients.

## Discussion

To our knowledge, this is the first study to directly compare the neurophysiological markers of cue-related attentional bias in IGD and OCD patients. The study participants with IGD exhibited increased LPP amplitudes in response to game-related pictures, indicating the enhanced salience of game-related cues. Interestingly, higher LPPs were observed in the IGD group, irrespective of specific game differences (i.e., League of Legends, FIFA, Sudden Attack). In addition, the patients with IGD did not exhibit LPP changes in response to OCD-related cues. Our results indicate that LPP would be a neurophysiological candidate marker for the diagnosis of IGD and craving of IGD.

The present study extends the findings of previous studies of addicted patients regarding attentional bias toward addiction-related cues to individuals with IGD, as evidenced by the increased LPP amplitudes in response to the visual presentation of online game-related pictures. Previously, for most substance use disorders, increased LPPs were consistently reported in relation to craving (20–22). Craving influences drug-seeking behavior (18) and has been associated with relapse in substance use disorders (14, 32). In a recent study of craving using LPP, increased LPP amplitudes for drug-related cues relative to non-drug-related cues were found in individuals with cocaine use disorder; however, the LPP changes were reversed from baseline to 6–month follow-up with treatment, wherein the extent of LPP reversal was correlated with decreased craving at follow-up (18). The ERP results obtained in the present study demonstrate an increased LPP amplitude in individuals with IGD compared with the amplitude measured in normal controls, in accord with a previous study of excessive computer gaming players (23) and studies of substance use disorder. However, our results failed to identify significant correlations with severity of craving in individuals with IGD. One aspect that could obscure the correlation with craving is that our subjects with IGD were not abstaining from gaming. Another aspect to consider is that for individuals with IGD, exposure to real-life cues often occurs multi-modally rather than only through a visual modality. A recent review noted that correlations between neural cue reactivity and clinical covariates are significantly more powerful for multisensory cues than for unisensory cues (8). However, previous EEG studies of individuals with IGD observed a pattern for resting-state EEG that was distinct from the pattern observed for individuals with substance use disorder. A previous study by our group found that lower absolute beta power may be a trait market of IGD, whereas higher absolute delta power has been observed for alcohol use disorder (33). Taken together, the results confirm attentional bias is present in IGD in response to game-related cues using the ERP paradigm. However, further studies are required to directly compare LPPs between individuals with IGD and those with substance use disorder.

Our results revealed no difference in LPPs according to differences in the cues of specific games (League of Legends, FIFA, Sudden Attack). Whether the pathophysiologies of IGD patients are heterogeneous in terms of, for example, the type of preferred internet game remains relatively unexplored. This study observed no differences among internet game types, which suggests that there are common underpinnings of the neurobiology of craving in IGD, regardless of subtle differences in game type. Several functional MRI studies have investigated the change in brain activation among individuals with IGD in response to the presentation of game-related cues, and abnormalities in the prefrontal cortex (PFC) have been consistently reported. In one of the first fMRI studies, game-related cues enhanced activation of the orbitofrontal cortex (OFC), nucleus accumbens, dorsolateral prefrontal cortex (DLPFC), and the caudate nucleus among individuals with IDG compared with the activation measured for a control group (15). Another study affirmed the activation of the DLPFC, parahippocampus and precuneus and found that the DLPFC and precuneus were correlated with subjective gaming urge under cue exposure (15). Meanwhile, a recent study that combined ERP and fMRI methods reported that enhanced source activity for emotional compared to neutral conditions in temporal, and frontal regions including ventromedial prefrontal cortex (34). The PFC processes emotion-related information (15) and is necessary for proper control, planning, and flexibility of behaviors (9). Hence, dysfunction in the PFC leads to the loss of flexibility to adjust the salience value of reward as a function of context and contributes to the bias of increased salience toward addiction-related cues, which may associate with craving. In this regard, the absence of game-specific differences in LPP in our results may suggest that a common mechanism, such as dysfunction of the PFC, is present in individuals with IGD.

Compulsive behaviors and unwanted, repetitive thoughts are characteristics of OCD (35), and converging neurobiological evidence demonstrates that dysfunction in the cortico-striato-thalamo-cortical circuit, particularly the OFC, underlies the pathophysiology of the disorder (36). Dysfunction of the OFC has been indicated in meta-analyses of both task-based and resting-state functional imaging studies (37), and structural changes in the OFC have also been consistently reported in previous MRI studies (37). Moreover, abnormalities in the OFC have been observed in unaffected relatives of patients with OCD (37, 38), suggesting a genetic influence on the circuitry of the OFC in the pathophysiology of OCD. The OFC subserves in the representation of reward and punishment and in inhibitory control; therefore, dysfunction of the OFC is associated with deficits in the function of response inhibition, set shifting and decision making in patients with OCD (37). It has thus been repeatedly proposed that dysfunction of the OFC related to the salience attribution system and inhibitory function also underlies the pathophysiology of addiction and OCD (39, 40). Volkow and Morales (9) proposed that the ventromedial PFC (including the OFC) becomes hyperactive in addiction when an individual is exposed to drugs or cues, increasing reward salience. In a previous study with cocaine abusers, activation of the OFC was associated with craving (39). However, prior studies of changes in cue-related ERP in OCD have been limited, although one study recently investigated the alteration of LPP in individuals with OCD (41). Paul et al. (41) reported enhanced LPPs in response to OCD-relevant pictures compared with LPPs measured in response to neutral pictures among OCD patients, and the LPP abnormalities persisted when cognitive distraction successfully reduced self-reported arousal. Accordingly, our results revealed higher LPPs in response to OCD-related cues than to game-related cues among patients with OCD. In our study, the subjective results suggested that individuals with IGD exhibited increased arousal to both game-related and OCD-related cues, and patients with OCD also presented concurrent arousal to both cues. The discrepancy between subjective concurrent arousal and group-specific LPPs response suggest that the LPPs appear to reflect the sustained increase in attention toward, and processing of salient stimuli instead of arousal *per se* (19, 41). In consistence with the previous LPP study, we could not find significant correlation between OCD symptom severity and LPP (41). It is partly because OCD could be better understood at a system or network level (42). Previous studies in OCD presented interconnected dysfunctions of error detection system as well as inhibitory control, and the former is associated with abnormalities of earlier ERP component such as error-related negativity (ERN) than LPP (43). Integrative approach using various ERP markers is needed in future studies to understand the pathophysiology of OCD.

A few studies have investigated compulsivity in individuals with IGD and reported inconsistent findings due to the use of different study methods. Our group previously reported no deficits in 15 patients with IGD using the Cambridge Neuropsychological Test Automated Battery (CANTAB) (7), but a study with 86 patients of IGD revealed dysfunction of cognitive flexibility (5). Moreover, other studies have reported deficits in cognitive flexibility using a cue-related go/no-go task (44). Fauth-Buhler and Mann (45) insisted that the inconsistency among findings related to compulsivity may be explained by whether game-related stimuli were employed and suggests that patients with IGD may have more reward-related problems than general problems with cognitive flexibility. Otherwise, impulsivity and compulsivity may contribute to the phenomenology of IGD in concerted manner as initial impulsivity followed by compulsivity in behavioral addiction (5). Further studies are needed to examine the effect of cognitive aberrance in IGD.

The limitations of the present study include the following. First, we did not observe a significant correlation between LPP changes and the clinical variables. Further investigation using multimodal cues or more real-life cues appears necessary. Another limitation of the study is that we only investigated cross-sectional differences between the groups. A longitudinal follow-up of LPP could reveal changes in cue-related attentional bias according to clinical prognosis. In addition, our samples were not gender-matched across 3 groups and HCs had high IQ with a standard deviation above the population mean. However, the strength of this study is that it is the first to directly investigate neurophysiological markers of cue reactivity in IGD and OCD.

To conclude, our study showed that LPP increased in response to disorder-specific cues in IGD and OCD (game-related cues and OCD-related cues), while subjective arousals were overlapped in response to both cues. Our findings indicate that LPP could be a neurophysiological marker for cue-related craving in IGD. These results provide additional neurophysiological evidence for understanding the mechanism of craving in IGD, further demonstrating the potential clinical utility of LPP in providing a marker for efficacy of IGD- related treatment, such as cue-exposure therapy.

## Author contributions

SK, MK, and J-SC were responsible for the study concept and design. SK, MK, and TL contributed to the acquisition of ERP data. MK and MP performed the ERP analysis. MK, SP, and TL contributed to the acquisition and analysis of clinical data. SK, J-SC, D-JK, and JK assisted with data analysis and interpretation of findings. SK drafted the manuscript. SK, SP, and J-SC provided critical revision of the manuscript for important intellectual content. All authors critically reviewed the manuscript content and approved the final version for publication.

### Conflict of interest statement

The authors declare that the research was conducted in the absence of any commercial or financial relationships that could be construed as a potential conflict of interest. The handling Editor declared a past co-authorship with the authors.
